# Genome Scale-Differential Flux Analysis reveals deregulation of lung cell metabolism on SARS-CoV-2 infection

**DOI:** 10.1371/journal.pcbi.1008860

**Published:** 2021-04-09

**Authors:** Piyush Nanda, Amit Ghosh

**Affiliations:** 1 Department of Biotechnology, Indian Institute of Technology Kharagpur, West Bengal, India; 2 School of Energy Science and Engineering, Indian Institute of Technology Kharagpur, West Bengal, India; 3 P.K. Sinha Centre for Bioenergy and Renewables, Indian Institute of Technology Kharagpur, West Bengal, India; US Army Medical Research and Materiel Command: US Army Medical Research and Development Command, UNITED STATES

## Abstract

The COVID-19 pandemic is posing an unprecedented threat to the whole world. In this regard, it is absolutely imperative to understand the mechanism of metabolic reprogramming of host human cells by SARS-CoV-2. A better understanding of the metabolic alterations would aid in design of better therapeutics to deal with COVID-19 pandemic. We developed an integrated genome-scale metabolic model of normal human bronchial epithelial cells (NHBE) infected with SARS-CoV-2 using gene-expression and macromolecular make-up of the virus. The reconstructed model predicts growth rates of the virus in high agreement with the experimental measured values. Furthermore, we report a method for conducting genome-scale differential flux analysis (GS-DFA) in context-specific metabolic models. We apply the method to the context-specific model and identify severely affected metabolic modules predominantly comprising of lipid metabolism. We conduct an integrated analysis of the flux-altered reactions, host-virus protein-protein interaction network and phospho-proteomics data to understand the mechanism of flux alteration in host cells. We show that several enzymes driving the altered reactions inferred by our method to be directly interacting with viral proteins and also undergoing differential phosphorylation under diseased state. In case of SARS-CoV-2 infection, lipid metabolism particularly fatty acid oxidation, cholesterol biosynthesis and beta-oxidation cycle along with arachidonic acid metabolism are predicted to be most affected which confirms with clinical metabolomics studies. GS-DFA can be applied to existing repertoire of high-throughput proteomic or transcriptomic data in diseased condition to understand metabolic deregulation at the level of flux.

## Author summary

Metabolic flux analysis in disease biology is opening up new avenues for therapeutic interventions. Numerous diseases lead to disturbance in the metabolic homeostasis and it is becoming increasingly important to be able to quantify the difference in interaction under normal and diseased condition. While genome-scale metabolic models have been used to study those differences, there are limited methods to probe into the differences in flux between these two conditions. Our method of conducting a differential flux analysis can be leveraged to find which reactions are altered between the diseased and normal state. We applied this to study the altered reactions in the case of SARS-CoV-2 infection. We further corroborated our results with other multi-omics studies and found significant agreement.

## Introduction

In several disease conditions, metabolic reprogramming serves as the epicenter for loss of homeostasis in the host cell[1–4]. Metabolism allocates biochemical resources to the cellular machinery. Disease progression either caused by internal disturbances in host cell or attack by pathogenic intruders require reallocation of biochemical resources[5, 6]. Apart from resource synthesis, metabolism has also been shown to reciprocally interact with other molecular events like epigenetic modifications and gene regulation[7–10]. The role of metabolism in several high incidence diseases like cancer[11, 12], tuberculosis[13] and neuronal disorders[14] have been described with the advent in high-throughput technologies to study metabolites and underlying reactions. Therefore, technological and methodological development in understanding metabolic states of cells will help us in gaining better understanding of disease mechanisms and strategies to design better therapeutics.

In the context of the current COVID-19 pandemic, several reports highlight the deregulation of multiple metabolic pathways in the host cell in response to SARS-CoV-2 infection[15–17]. Cases of altered lipid metabolism is observed in patients who were infected with SARS Cov between 2002 and 2003, 12 years after their recovery. Moreover, reports of altered tryptophan and lipid metabolism has been elucidated in clinical samples of COVID-19 patients. Understanding the metabolic alterations at a genome-scale will help us in gaining deeper insights into the mechanism of pathogenesis beyond immunological effects. With increasing evidence on the importance of metabolism in disease biology in particular the current COVID-19, it has become imperative to develop technologies to probe into activities of reactions that underlie metabolic pathways. Flux, which is defined as the reaction rate per unit biomass, directly reflects on the homeostatic balance of the cell. Disturbance of cellular homeostasis due to pathogenic intruders is clearly reflected in alterations in metabolic pathways. In this regard, Genome-Scale Metabolic Models (GEMs) have been leveraged to understand flux distributions[18–20] using tools like Flux Balance Analysis (FBA). Through years, GEMs of human cell lines have been used for understanding progression of disease, host-pathogen interactions and up to neurological disorders[21, 22]. Algorithms like GIMME, iMAT, MBA, INIT/tINIT have enabled us to incorporate gene expression or proteomics data into the model (GEMs) and hence helping the construction tissue or cell or condition specific GEMs[23]. This enables us to enlist reactions which are differentially altered between normal and diseased state. Highly curated Human GEMs like Recon3D[24] and HumanGEM[25] have incorporated finer details about the human metabolism. They have been validated under multiple experimental conditions and have shown to be representative of the human metabolism in both normal and diseased conditions.

Despite the advancement in metabolic modeling, most of the work has focused on elucidating altered reactions based on differentially incorporated reactions in condition-specific metabolic models. Such an approach, allows us to map the effects on gene expression or enzyme production on reactions qualitatively. Our ability to find the degree of alterations at the level of fluxes in such context specific models will enable us to understand the activities of different pathways in the cell. Fluxes are a consequence of interactions between several reactions, the structural features of the metabolic pathways and the nutrient uptake rates[26]. Limited analysis has been done to leverage the power of metabolic flux analysis to probe into differences in fluxes between diseased and non-diseased state. The altered reactions calculated using the differences in flux would also enable us to conduct enrichment analysis of pathways and pin point specific metabolic modules altered between two conditions i.e. normal and diseased. Although computational methods exists to infer metabolic changes using relative gene expression[27] or transcriptome data[28] data but none infer differences at the level of flux directly. FBA, while being a powerful tool in microbial systems biology, requires a strict objective function such as specific growth rate[29]. This restricts its usage in the case of GEMs of higher eukaryotes where it’s difficult to define single cellular objectives. Approaches like Flux Variability Analysis (FVA)[30] and flux sampling[31] alleviate this problem by allowing us to obtain the limits of the solution space. These tools have not been put into a statistical framework to allow us to conduct differential flux analysis and pathway enrichment studies as we do for gene expression.

While such steady state approaches, allows us to predict reaction fluxes under given conditions, it doesn’t speak much about the mechanism of flux regulation. To understand the regulation of reactions, it becomes imperative to conduct integrative analysis of metabolic flux analysis with other omics approaches. Omics datasets such as protein-protein interaction networks and phosphorylation variations can help us in inferring the prospective causative factors behind alterations in metabolic flux. Therefore, our ability to develop pipelines for integrated analysis of drivers (alterations in post-translational modifications of enzymes) and effects (fluxes) will enable us to obtain better insights into disease mechanisms.

In this study, we developed a Genome-Scale Differential Flux Analysis (GS-DFA) that leverages flux sampling and stringent statistical framework to report affected metabolic modules in diseased conditions. We developed a context-specific integrated GEM of SARS-CoV-2 infected NHBE cells leveraging the publicly available gene expression data. We apply the GS-DFA to the context specific GEM of normal human lung cells or tissues (NHBE and Lung Biopsy) and SARS-CoV-2 infected human lung cells. We show that several expected pathways are affected and significant number of altered pathways have associations with viral proteins through PPIs or/and altered phosphorylation landscape. Several pathways such as fatty acid oxidation, arachidonic acid metabolism (eicosanoid metabolism), fatty acid biosynthesis, cholesterol biosynthesis and amino acid metabolism is affected in diseased state. Some of these pathways i.e. arachidonic acid metabolism are associated with the production of immune-regulatory molecules like Leukotriene and Prostaglandins which mediate cell-to-cell signaling and inflammatory response management[32]. SARS-CoV-2 proteins interacts with multiple enzyme subunits driving the reactions we enlisted to be differentially altered. This indicates a mechanism by which the virus possibly reprograms the metabolism of the host cell in order to facilitate its growth and survival.

## Results

### Stoichiometric model of SARS-CoV-2 biomass

**Fig 1:**
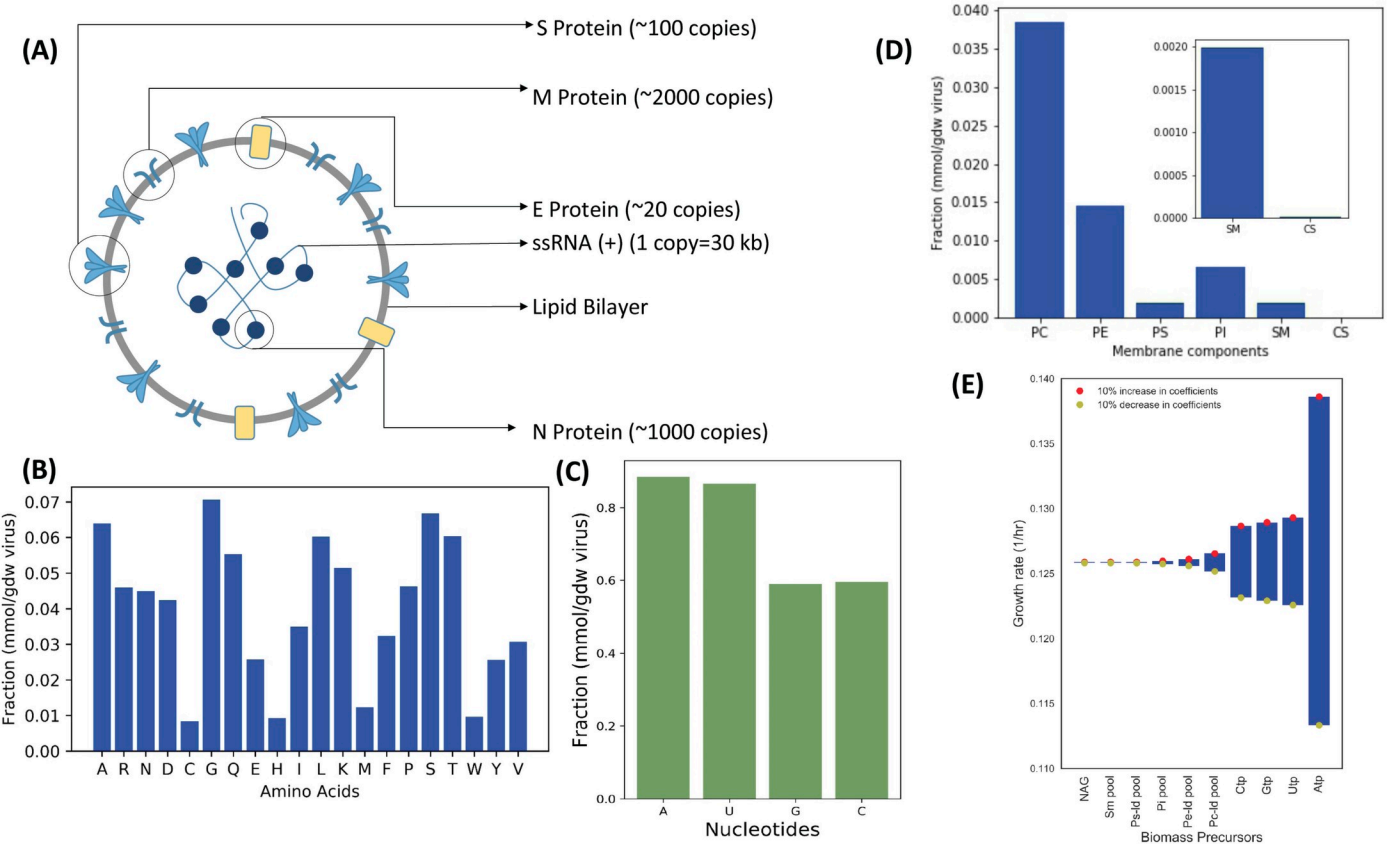
Reconstruction of biomass equation (VBOF) of SARS-CoV-2 virus from its stoichiometric make-up. (A) The stoichiometry of various proteins, nucleic acid and their locations in the SARS-CoV-2 virus. The molar composition of constituent molecules in lipid fraction (B), nucleic acid (C) and proteins (D). PC-Phosphatidylcholine; PE-Phosphatidylethanolamine; PS-Phosphatidylserine; PI-Phosphatidylinositol; SM-Sphingomyelin; CS-Cholesterol. (E) Sensitivity analysis of growth rate of SARS-CoV-2 with respect to coefficient (fractional composition) of biomass precursor. The coefficients are varied by ±10% and y-axis shows growth rates simulated by Flux Balance Analysis (FBA).

Flux balance analysis requires a biomass equation which would serve as an objective function for the underlying linear optimization problem[26]. The biomass equation describes the accurate stoichiometry of the biological system in question based on the molecular make up of its constituent proteins, nucleic acids, carbohydrates and lipids. We sought to define a similar biomass equation for SARS-CoV-2. Integrating the same into the GEMs of the human lung cells would allow us to analyze the host-virus metabolic interaction. Each virus particle comprises of a single stranded positive sense RNA, proteins at the membrane and in complex with the RNA, carbohydrates studded on some of the membrane proteins and a lipid bilayer[33] (Fig 1A). Leveraging the recent literature data on copy number of the macromolecules and their chemical composition, we constructed a virus biomass objective function (or biomass equation)-Cov2VBOF. We used spike-protein copy number per virion as a basis to calculate the absolute copy number of proteins on the virus leveraging proteomics datasets. Here, the fractional molecular contribution of each amino acid was calculated solely from the copy number of proteins and their amino acid composition (Fig 1B). Similarly, we used information on translational efficiency over the viral genome[34] and the absolute copy number of the proteins, to calculate the mRNA levels resulting from the viral genome. Several studies show the low GC content of the virus is intrinsically linked to a strategy to avoid recognition by the innate immune system. Particularly, ZAP proteins recruit themselves to CpG enriched regions and trigger host machinery to degrade the viral genome[35]. We therefore observe a lower fraction of GC in the SARS-CoV-2 genome and the corresponding transcriptome (Fig 1C).

Phosphatidylcholine and Phosphatidylethanolamine occupied the highest lipid molar fraction in the membrane whereas cholesterol occupied the lowest molar fraction (Fig 1D). The biomass equation has 44 biomass components including all 20 amino acids, 4 nucleotides, lipid and cholesterol, carbohydrates along with ATP required for polymerization of the macromolecules. N-Acetyl Glucosamine (NAG) is a molecular coating on the Spike protein of SARS-CoV-2 which allows the virus to mask the potential epitopes against which the host cell can develop anti-bodies[36]. The SARS-CoV-2 infection activates the NAG biosynthesis pathway in order to meet the viral demands. To show the importance of biomass precursors towards fitness of the virus, we conducted a biomass sensitivity analysis. Here, we tried to capture the effect of small fluctuations (~10%) in coefficient of biomass precursor on the specific growth rate prediction by FBA (Figs 1E and S4). We show that the nucleotides (highest), lipids and NAG (lowest but non-zero) affect the specific growth rate the most. Such an analysis allows us to rank the biomass precursors based on their relative importance on the fitness. As a significant fraction (Fig 1D) of SARS-CoV-2 biomass is based on its nucleic acid, it is likely that fluctuations in their components will affect the growth rate the most. The components of lipid membrane also affect the growth rate of the virus significantly as unlike amino acids, they are de novo synthesized in the host cell. Similarly, N-acetylglucosamine is synthesized de novo to serve as a carbohydrate coating on the surface proteins of SARS-CoV-2. This analysis allows us to understand the biomass components of SARS-CoV-2 whose variation might affect the growth rate of SARS-CoV-2 severely.

**Fig 2:**
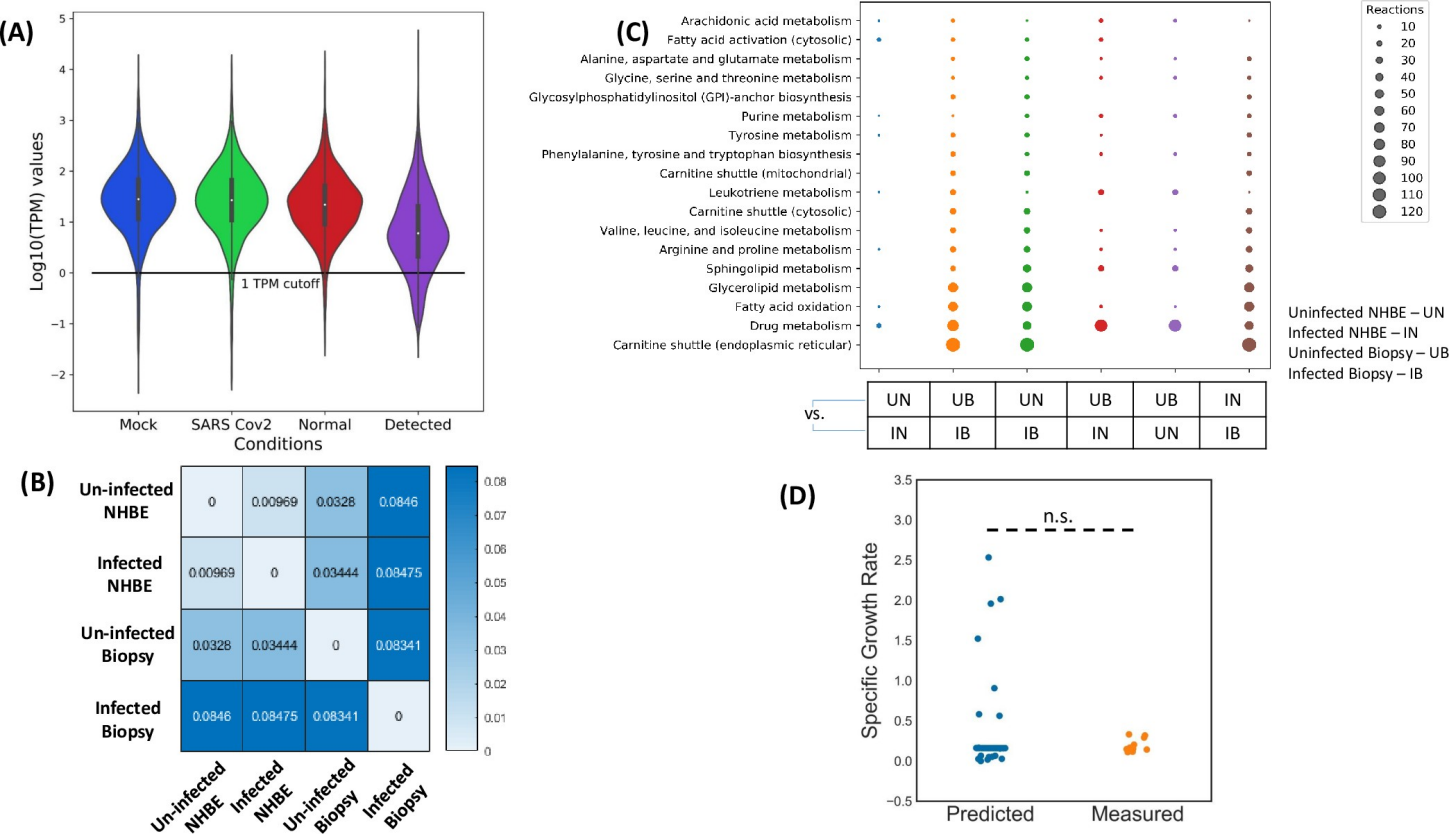
Generation of context-specific models of SARS-CoV-2 infected cells and normal cells using gene-expression data and tINIT algorithm. (A) The distribution of TPM normalized gene expression in Mock NHBE cells, SARS-CoV-2 infected NHBE cells, Normal Lung Tissue (From Biopsy) and Infected Tissue (From Biopsy). (B) The Hamming distance between all the generated context specific models based on the differences in reactions in the model. (C) The pathways over-represented while comparison between models. The reactions over-represented in each comparison are present exclusively in one of the models. (D) The agreement of model simulated specific growth rate of the virus with the experimental reported specific growth rate. n.s. stands for non-significant differences as estimated by t-test. The model simulated growth rate was calculated under ±5% uncertainty in biomass composition and uptake rates. The experimental growth rates were derived from Bojkova et al. (Nature), 2020.

We further integrated the Cov2VBOF into the context specific models of NHBE cells in infected state. We showed the Cov2VBOF helps in accurately simulating the growth kinetics of the virus upon infection. By varying the coefficient of each precursor by 50% (an extreme variation), we were able to show the predicted growth rate shows no significant difference with the experimental reported growth rates of the virus (p>0.05, t-test) (Fig 2C). As several different methods were used to measure the experimental growth rate[37] in literature, the constructed biomass equation for SARS-CoV-2 confirms with the realistic chemical composition of the virus.

### Integration of transcriptomic profile in Human GEM

In the case of mammalian cells, it becomes particularly important to constrain the reactions in the generalized HumanGEM metabolic model based on expression levels in the cells to define the metabolic model of a given cell type. This eliminates reactions that are lowly expressed in the specific cell/tissue while keeping reactions having high expression or are required for basic metabolic functionality. We used task based INIT[38] (Integrative Network Inference for Tissues) to define NHBE cell and Lung Biopsy context specific metabolic models with and without viral infection. The tINIT algorithm doesn’t require a fixed objective function, allowing for an unbiased integration of gene expression data. The TPM normalized gene expression data[39] (Fig 2A) was integrated into the model as described in material and methods. The NHBE background context specific metabolic models were named as iNHBE and iNHBECov2 for normal NHBE and infected NHBE respectively. Similarly, the lung biopsy specific metabolic models were named iLungsTissue and iLungsTissueCov2. Majority of altered reactions in infected NHBE cells (iNHBECov2) compared to normal NHBE (iNHBE) belonged to arachidonic acid metabolism, fatty acid oxidation, fatty acid activation and few amino acid biosynthesis pathways of arginine, proline and tyrosine (Fig 2C). Deregulation of arachidonic acid metabolism has been reported earlier to be associated with coronavirus infection. Linoleic acid to arachidonic acid metabolism axis is one of the most perturbed reactions as reported through metabolomics analysis of HCov-229E infected cells[40]. We also observe a perturbation in Leukotriene pathways between the iNHBE and iNHBECov2 cells (Fig 2C). Arachidonic acid is the precursor for the production of leukotrienes and prostaglandins through oxidation[32]. Leukotrienes are also important cell signaling molecules which are involved in both autocrine and paracrine signaling[32]. More particularly, leukotrienes are important class of molecules that have chemotactic action of migrating immune cells. Prostaglandins are mediators of inflammatory responses in the cell[32]. Similarly, between iLungsTissue and iLungsTissueCov2, apart from pathways affected as in the case of NHBE, we observed differences in reactions belonging to carnitine shuttle (endoplasmic reticulum and mitochondria), glycerolipid and sphingolipid metabolism (Fig 2C). There were also differences in several amino acid metabolism pathways of glycine, serine, alanine and aspartate. The differences in prediction of affected reactions in the case of NHBE cell context specific GEMs and Lung Biopsy context specific GEMs could be due to the heterogeneity of tissue samples. The affected reactions in the case of lung tissue biopsy presents the metabolic alterations at the level of tissue resulting from metabolic interactions among the constituent cell types. NHBE cells are laboratory adapted epithelial lung cells and the context specific GEMs corresponding to cellular level response to the infection. We further compared the context specific GEMs of NHBE cells and the lung tissue biopsy to elucidate fundamental differences in metabolic state both in infected and uninfected conditions (Table 1). We used Hamming distance as a proxy for quantifying the differences in the metabolic architecture of the context specific models (Fig 2B). Carnitine shuttle pathway, which activates fatty acids and transports them for fatty acid beta-oxidation, is a major affected pathway in the case of lung tissue biopsy of infected patients compared to normal (Fig 2C). This pathway is not particularly observed to be an affected pathway in the case of NHBE cells. We hypothesize the differences might be due to metabolic interactions at the level of tissue and contribution of heterogeneous cell types.

**Table 1:** Number of reactions, metabolites and genes in each of the model generated for differential flux analysis.

	iNHBE	iNHBECov2	iLungsTissue	iLungsTissueCov2
**Reactions**	7270	7255	7288	6528
**Metabolites**	5287	5276	5300	4977
**Genes**	2746	2737	2732	2263

The Cov2VBOF was leveraged to measure the specific growth rate of the virus in the context specific model of the infected NHBE cell. We used Flux Balance Analysis (FBA) on the context specific model constrained by HAM media using Cov2VBOF as an objective function. We repeated the FBA for several perturbations in the Cov2VBOF (to account for effect of uncertainty in biomass composition on growth rate) and small alterations in the uptake rates of nutrient in the infected conditions (Accounting for effect of uncertainties in uptake rates of media components). We compared the predicted specific growth rate to the experimental growth rate as reported in earlier[41]. We could find no significant differences between the predicted and experimental growth rate (p>0.05, t-test) (Fig 2D). This is a strong evidence for the validity of the constructed virus biomass objective function. It is to be noted that the specific growth rate estimate here is the approximate upper bound. This would be the observed growth rate if all of the resources are being diverted towards the growth of the virus.

**Fig 3:**
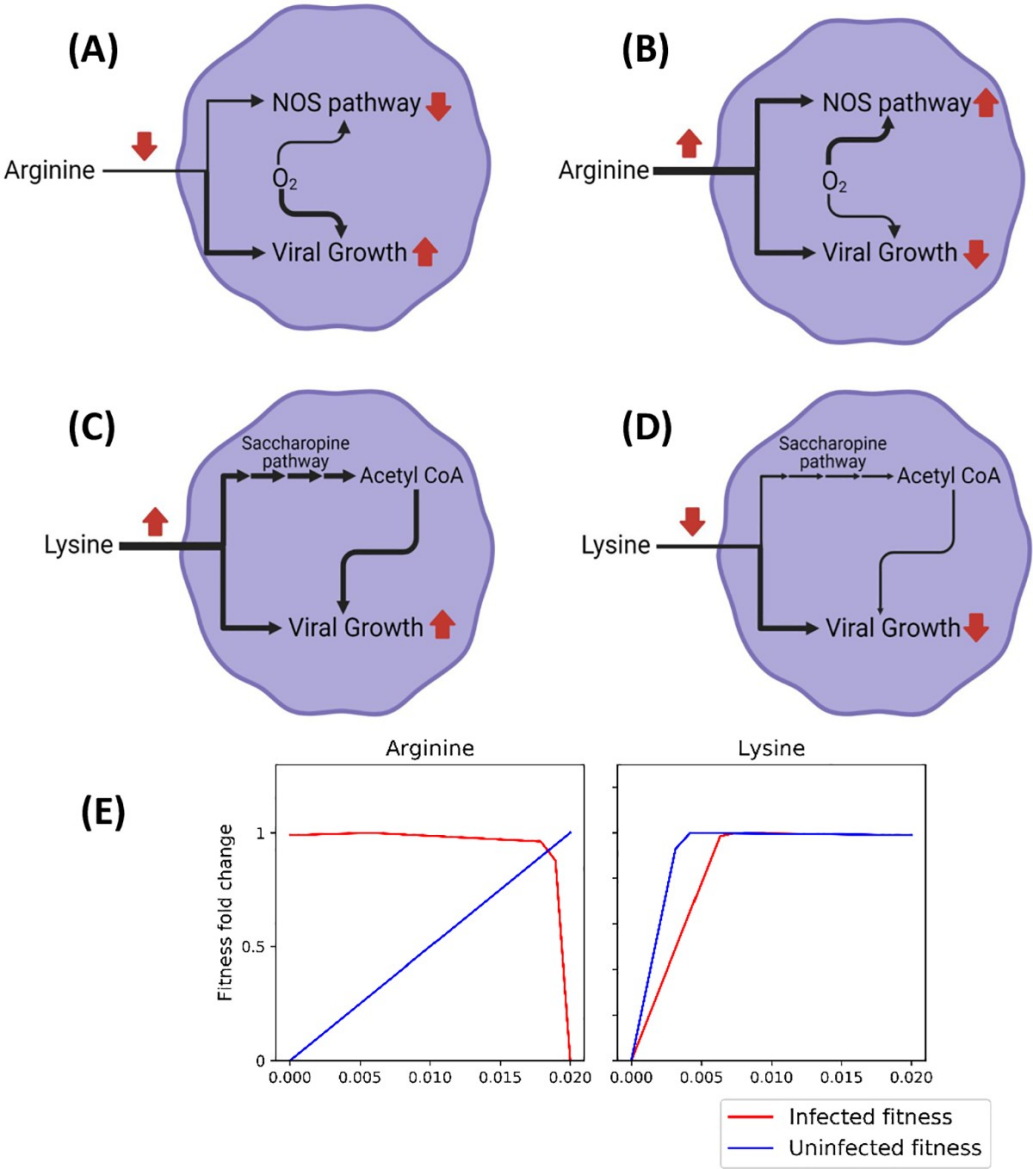
(A) and (B) show the metabolic condition for low and high arginine uptake rates respectively. A high arginine uptake increases the flux through NOS (Nitric Oxide Synthase) pathway which scavenges oxygen away from aerobic metabolism and impairs viral growth. (C) and (D) show the metabolic states for high and low lysine uptake. A low lysine uptake reduces the flux through lysine degradation pathway (Saccharopine pathway) and reduces the supply for Acetyl-CoA required for viral growth. (E) The robustness of specific growth rate of SARS-CoV-2 with respect to changes in specific uptake rates of arginine and lysine. Refer to S3 Fig for robustness analysis with respect to specific uptake rates of all nutrients. Fig 3A–3D were created using BioRender.com.

Furthermore, we tried to capture the effect of specific uptakes rates (mmol/gdw/hr) of nutrients on the fitness of the virus in infected cells through phenotypic phase plane analysis. Such an analysis allows to visualize the effects of alterations in uptake rates (Phenotype 1) on the growth of the virus i.e. fitness (Phenotype 2) (S3 Fig). It was interesting to note that increased uptake rates of arginine severely affects the fitness of the virus while it supplements the growth of the normal NHBE cells. A higher uptake rate of arginine i.e. beyond the cellular need, pushes the flux through the Nitric Acid Synthase (NOS) pathway (Fig 3A and 3B). The Nitric Acid Synthase pathway consumes arginine, NADPH and oxygen to function. Compared to metabolic model with normal uptake rates of arginine, NOS pathway in higher arginine uptake, scavenges oxygen from the cytosol leading to impairment in aerobic metabolism in cells. This leads to detrimental effects on growth of the virus (Fig 3E). We also noted the effect of decreased uptake of lysine on growth of the virus (Fig 3C and 3D). Interestingly, a reduced uptake of lysine impairs the growth of the virus. Lysine is normally catabolized through the saccharopine pathway and fed into the TCA cycle through Acetyl-CoA. Reduced lysine uptake brings down the supply of Acetyl CoA leading to a retardation in viral growth (Fig 3E). Amino acid transport in the cells are mostly driven by facilitated diffusion through transporters. Therefore, the concentration of amino acids in the cellular environment would dictate the uptake rate of amino acids. Similar approach of supplementing or depleting amino acids have been used as therapeutic option for treating of cancer and other diseases[42].

Context specific metabolic models of NHBE cells and lung biopsy helped us to catalogue altered metabolic reactions between normal and infected condition. The network properties of the metabolism and the gene-protein-reaction association captured by the HumanGEM model combined with the gene expression data allowed us to analyze altered metabolic phenotypes in diseased state. These models can be further leveraged to reveal more biological insights into the metabolic state of the diseased cell using powerful tools of metabolic flux or network analysis.

### Differential flux analysis for hunting altered reactions in SARS-CoV-2 infected cell

**Fig 4:**
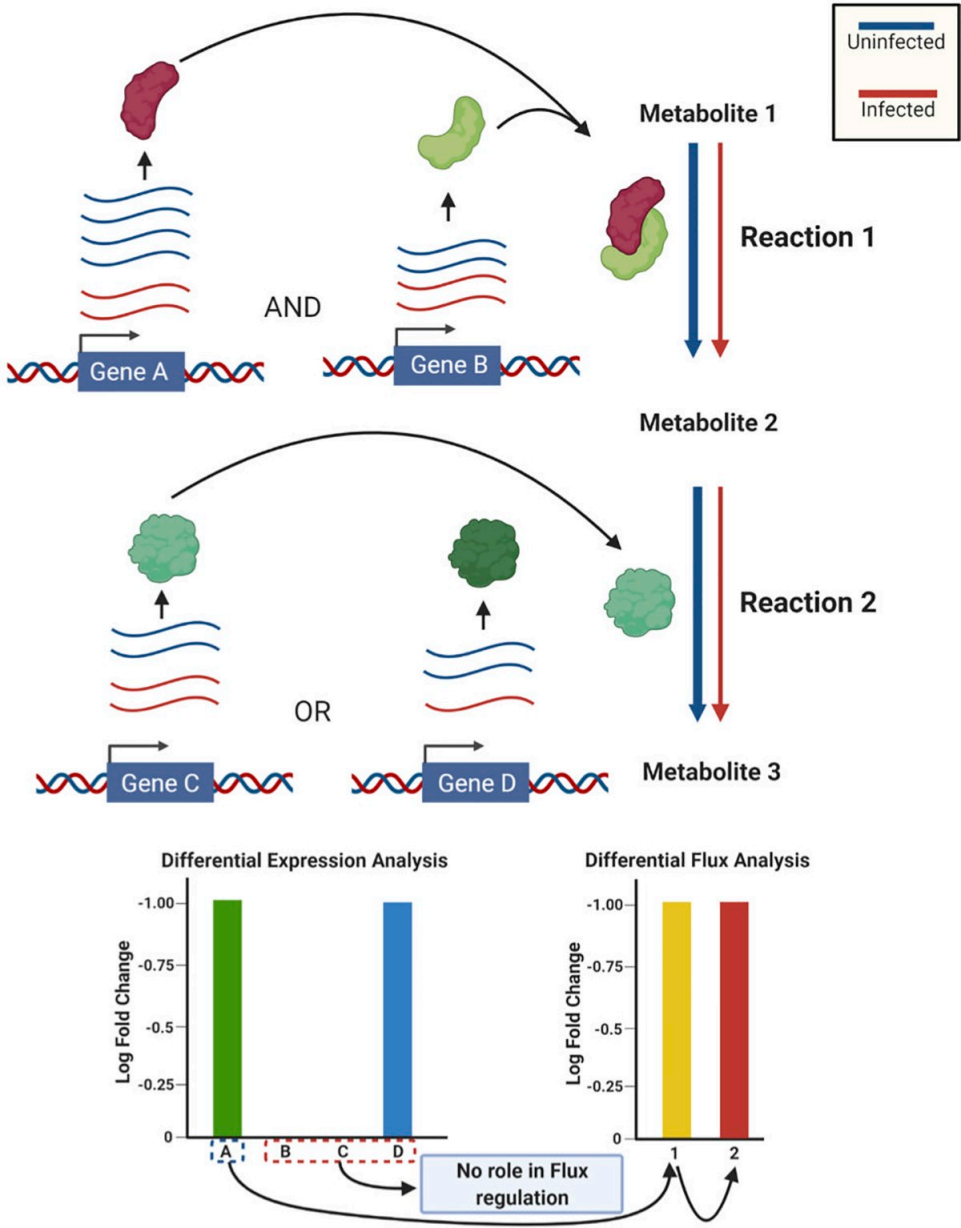
Boolean relation between activity of enzymes and the gene encoding the proteins in the complex makes the prediction of flux from expression difficult. Gene A and Gene B encodes subunits of the enzyme which catalyzes ‘Reaction 1’. Gene C and Gene D encode two alternative isozymes which catalyzes ‘Reaction 2’. The width of the arrow for ‘Reaction 1’ and ‘Reactions 2’ represents the level of flux. The black arrow is in the direction of cause to effect. A fold change in the expression of A is sufficient to change the flux through reaction 1 which further changes through reaction 2. All the enzymes are assumed to have their concentration above the threshold. (Created with BioRender.com).

Integration of gene expression data into the highly curated HumanGEM pruned non-essential reactions while enriching important reactions. This helped us to generate context specific models of normal and diseased cells. We further aimed at understanding the alterations in metabolic flux in an infected cellular state. Such an analysis would pinpoint specific reactions which show altered behavior in diseased state. While inferring altered metabolic pathways have been conventionally done using differential gene expression analysis, we show the limitations of such analysis. The presence of either isozymes (multiple enzymes having same function) or multi-subunit complex (multiple subunits contributing collectively towards a function) makes it difficult to infer the effects of altered gene expression on metabolism[23]. It is very well known that alterations in levels of enzyme facilitating the rate limiting step can lead to changes in flux distributions in the downstream reactions. To illustrate why gene expression might not be sufficient to predict the causative factors behind the alterations in metabolism, we consider a hypothetical linear pathway (Fig 4). Gene A and Gene B encode two separate subunits of an enzyme complex catalyzing ’Reaction 1’. Gene C and Gene D encode two alternate copies i.e. isozymes catalyzing ’Reaction 2’. It is to be noted that both Gene A and Gene B are necessary for ’Reaction 1’ to take place. For ’Reaction 2’, either Gene C or Gene D is required as they can compensate for each other. ’Metabolite 2’ is not diverted towards any other reaction and therefore, the flux through ’Reaction 1’ equates to that through ’Reaction 2’. A disease perturbation downregulates Gene A and Gene D by two fold. A decrease in copy number of ’Gene A’ brings down the normal level of the enzyme complex. In this case of ’Gene D’, the level of the enzyme doesn’t change as a threshold concentration of ’Gene C’ is already present (Gene C doesn’t undergo any downregulation). Gene expression analysis would show changes in levels of ’Gene A’ and ’Gene D’ as the causative factors behind alteration in metabolism. In reality, ’Gene D’ didn’t impart a change in the flux through the reaction it was catalyzing as its absence was compensated by ’Gene C’. Downregulation of ’Gene A’ in this case was sufficient to bring down the flux through ’Reaction 1’ reducing the supply of ’Metabolite 2’. This resulted in a corresponding decrease in flux through ’Reaction 2’. As GS-DFA uses flux to quantify the metabolic alteration, it provides the cause-effect relationship between the gene expression and metabolic flux, allowing us to design better therapeutics to restore the abnormality. From a therapeutic point of view, targeting ’Gene D’ wouldn’t restore the homeostasis as it not a causative factor whereas targeting ’Gene A’ would.

Metabolic flux analysis allows us to understand the flux distribution in a model under a given set of condition. While tools like FBA enable us to obtain possible flux values for reactions throughout the metabolic model, it doesn’t guarantee uniqueness of the solution and necessitates an objective function[23]. Flux variability analysis and flux sampling allow us to capture the extent of the flux solution space[30]. Hence, the information of the solution space can be used to understand the quantitative features of metabolic phenotypes. We were further interested in comparing the flux solution spaces between two conditions (normal vs. diseased) and highlight reactions that have altered flux. But given the lack of precision in metabolic flux analysis, stringent statistical filtering is important for such an analysis.

**Fig 5:**
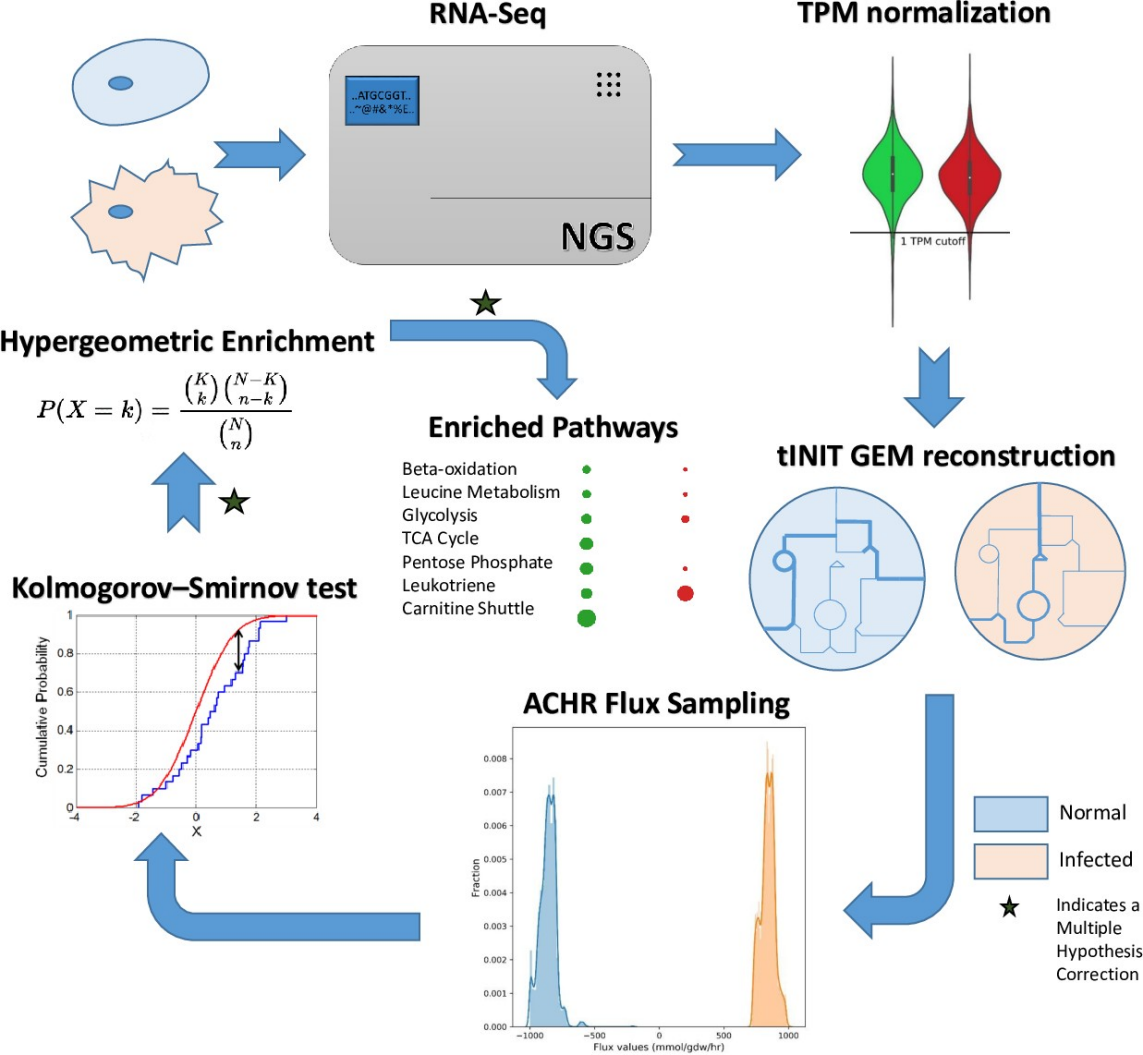
The pipeline for GS-DFA beginning from RNA-seq of infected and normal cell line, followed by construction of context specific models, flux sampling, reaction filtering and over-representation analysis of pathways. The ‘star’ symbol indicates a multiple hypothesis correction step through Benjamini-Hochberg False Discovery Rate. A two-sample Kolmogorov-Smirnov test is used to differentiate between the probability distribution of flux between diseased and non-diseased state.

Flux sampling allows us to screen through all the possible flux values and generate a probability distribution of the values that a given reaction can undertake[31]. This can be directed for comparing the flux values distribution between a normal states vs. diseased states. We developed a pipeline (Fig 5) that takes the context specific models of NHBE and Lung Biopsy samples in both normal and diseased state, performs a differential flux analysis to reveal altered reactions. The pipeline leverages Kolmogorov-Smirnov test to compare the flux probability distribution for given reactions between two conditions. The altered reactions having statistical significant differences in the distributions and a high fold change (F.C. cutoff ~ Flux 1 is 10 times Flux 2) are used for reaction enrichment analysis among the metabolic subsystems present in the model. A positive flux change refers to an upregulation of metabolic flux through the reaction whereas a negative flux change refers to a potential downregulation. The effect size (flux change) for each of the affected reactions in an over-represented pathway helps to decide the direction of impact with respect to the uninfected cells (S2 Dataset).

**Fig 6:**
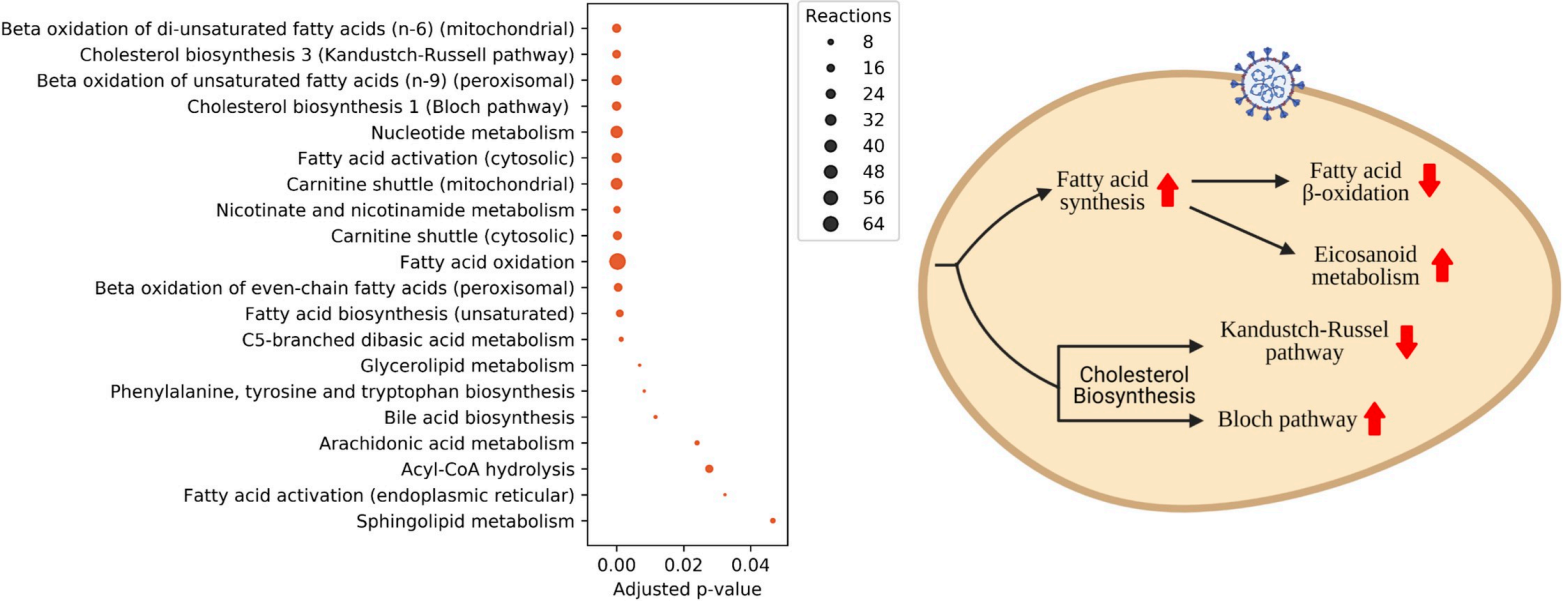
GS-DFA reveals altered pathways in infected cells and post-translational modifications in infected cells are enriched in altered reactions. (A) Enriched pathways (subsystems) in the SARS-CoV-2 infected NHBE cells as revealed by GS-DFA analysis. The presented reactions have flux change (as described in materials and methods) greater than 0.82 and have adjusted p-value less than 0.05 (B) Some of the pathways altered in the SARS-CoV-2 infected cells that could have therapeutic relevance (Created with BioRender.com).

For NHBE cell context specific models i.e. iNHBE and iNHBECov2, cholesterol biosynthesis (Kandutsch-Russel Pathway and Bloch pathway), fatty acid oxidation, beta-oxidation of di-unsaturated fatty acids (mitochondria), fatty acid activation, carnitine shuttle pathway (mitochondria and cytosol), arachidonic acid metabolism and few amino acid biosynthesis pathway were severely altered between the normal vs. diseased state (Fig 6A). Majority of the altered reactions belonged to fatty acid oxidation pathway. Fatty acid oxidation is severely impaired in the infected cells. We see a reduced flux through majority of fatty acid oxidation reactions in the mitochondria compared to uninfected cells. This would also indicate a higher accumulation of free fatty acids which cannot be oxidized in the mitochondria. Interestingly, reports show an increased profile of multiple fatty acids in clinical samples in infected cells[15]. There is an upregulation and higher flux through fatty acid biosynthesis in the infected cells compared to the control. It is very well known that virus ramps up the fatty acid synthesis to support its growth i.e. synthesis of viral membranes[43]. For all the reactions in the fatty acid biosynthesis pathways, we see a high flux through them. Arachidonic acid metabolism, which has been earlier reported to be one of the severely altered pathways in HCov-229E, also showed altered flux for several reactions[40]. There is an increased flux from arachidonic acid to eicosanoid metabolism. Eicosanoid metabolism leads to the production of leukotrienes and prostaglandins, both of which are important immune inflammatory molecules. It is not surprising as inflammatory storm is one of the cornerstone of COVID-19 infection. Increased synthesis of inflammatory molecules could be the host response to the infection. The role of leukotrienes in trigger inflammatory reactions has been highly discussed in the context of COVID-19[44]. Beta-oxidation of fatty acid has also been discussed to play a significant role in the progress of the infection(41). Cholesterol biosynthesis comprises of two parallel routes i.e. Kandutsch-Russel Pathway and Bloch pathway. In our predictions, we find Bloch pathway to be heavily upregulated where as Kandutsch-Russel pathway is downregulated and has significantly lower flux in infected cells. The final production of Kandutsch-Russel pathway is 7-Dehydrocholesterol which feeds into the vitamin D biosynthesis pathway. Interestingly, the role of vitamin-D, which modulates immune inflammatory action, in alleviating the COVID-19 infection has been discussed earlier. An impaired synthesis of vitamin-D due to reduced flux through its precursor pathway i.e. Kandutsch-Russel pathway could result in deregulation of inflammatory pathways in the cells. There were also several differences in beta-oxidation pathways of di-unsaturated and fatty acid activation. Of the amino acid biosynthesis pathways, aromatic amino acid pathway (Phenylalanine, Tyrosine and Tryptophan) was altered at the level of flux between normal and diseased state. Interestingly tryptophan metabolism has been reported to be altered during SARS Cov2 infection through metabolomics studies(15). Alterations in tryptophan metabolism has been implicated in several viral diseases in previous studies and has been proposed as an important therapeutic target. Kynurenine, which is a product derived from tryptophan by the activity of IDO1 (indoleamine 2,3 dioxygenase 1), is an important immune-regulatory molecule. Defects in IDO1 has been shown to be correlated to IL6 induced inflammation in cells(15). There is also significant alterations in nucleotide metabolism. This could results from high demand for RNA synthesis in the host to support the complicated transcriptional readout in the virus and the synthesis of the ssRNA while suppressing the DNA synthesis. An increased activity of carnitine shuttle responsible for fatty acid beta-oxidation is also observed in the altered reactions.

Several pathways highlighted in this analysis (Fig 6B) are also the ones which are implicated (S2 Dataset) in SARS-CoV-2 or in some cases SARS-CoV. They could serve as potential therapeutic targets. While metabolic flux analysis highlights reactions that are altered based on their flux value distribution, it doesn’t give us any information about the molecular mechanistic principles driving such changes. Thus, it becomes imperative to leverage other ‘layers’ of omics data to understand the molecular modifications on enzymes leading to reduced or enhanced capacity for flux.

### Protein-protein interactions and altered phosphorylation potentially mediate metabolic changes

**Fig 7:**
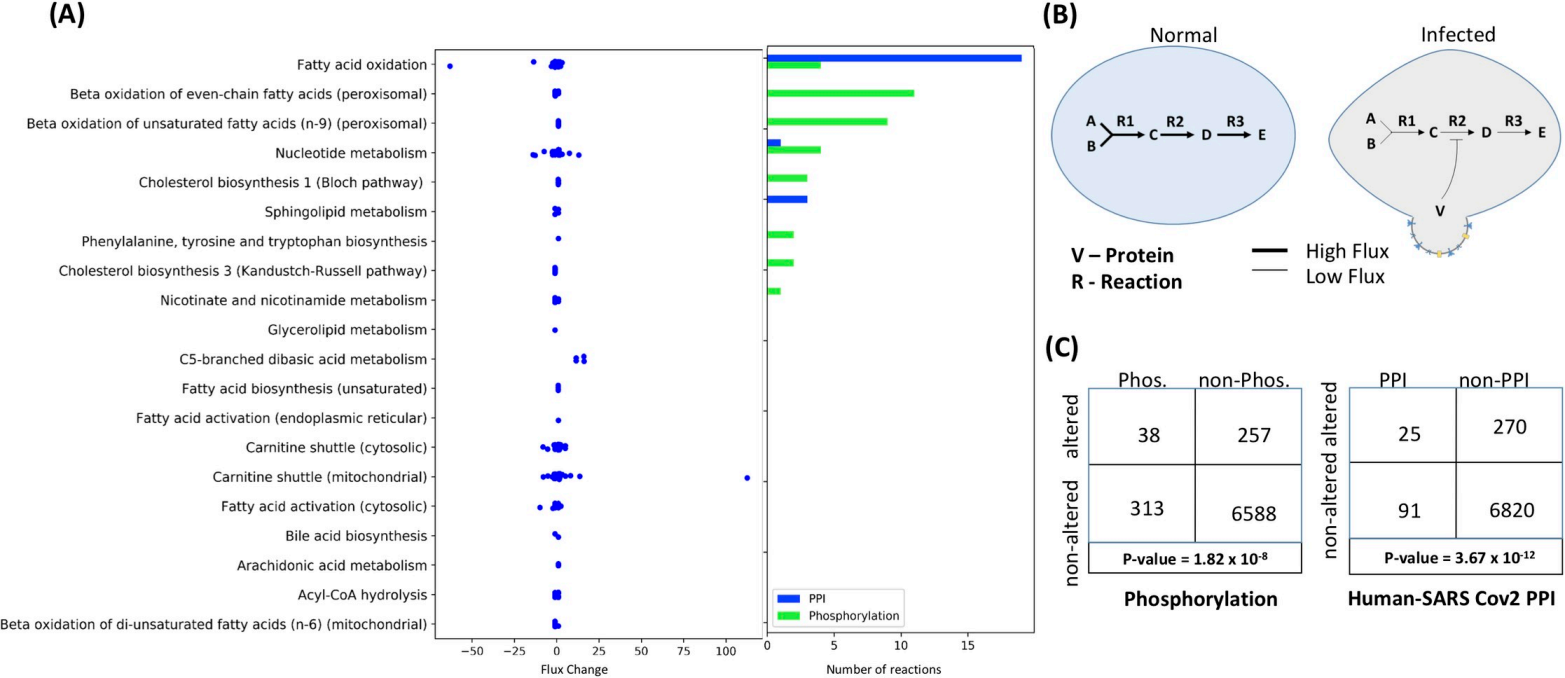
(A) A pathway wise description with the flux changes of the constituent altered reactions in the pathways along with the number of such reactions affected by differential phosphorylation and protein-protein interactions with viral proteins. (B) The prospective mechanism of flux regulation by post-translational modification or allosteric effects on enzymes mediated by viral proteins. (C) The enrichment of differentially phosphorylated enzymes and enzymes interacting with viral proteins in the set of altered reactions identified by GS-DFA.

It has been reported earlier that most RNA viruses modulate the metabolism and physiology of the host cell through post-translational modifications rather than transcriptional changes[45]. We were therefore interested to check if the enzymatic complex driving the reactions affected at the level of flux in diseased state are post-translationally modified. We leveraged the data on binary protein interaction network between host and SARS-CoV-2 proteins[46], and differential phosphorylation[47] dataset for this purpose. In our proposed model, we suspect several viral proteins interact with metabolic machinery of the host cell and press changes either through i) Allosteric modifications ii) Phosphorylation/Dephosphorylation induced effects (Fig 7B). We did a cross interaction analysis between the altered metabolic pathways and the post-translational modifications (Fig 7A and 7C). Surprisingly, several proteins interaction networks existed between the viral proteins and the enzymes involved in fatty acid oxidation, beta-oxidation of even chain fatty acids, nucleotide metabolism and sphingolipid metabolism. These are some of the severely affected pathways highlighted through our differential flux analysis. Furthermore, overlaying the differential phosphorylation dataset on our metabolic flux analysis, revealed several reactions belonging to beta-oxidation of fatty acids (peroxisomal), fatty acid elongation and omega-3 fatty acid metabolism have modified phosphorylation state between the normal and diseased condition (Fig 7A).

This directly indicates the modification of host metabolism induced by the viral proteins through post-translational modification. We also acknowledge the presence of complicated pathways of modifications through host signaling pathways which we might have missed in our analysis. Phosphorylation and allosteric modification are two significant pathways through which metabolic machinery is regulated at the post-translational level. Inhibitors against the viral proteins mediating such molecular modifications can be used as a therapeutic option to restore metabolic homeostasis in the cell. Nevertheless, fatty acid metabolism appears to be one of the most affected pathways in the host which can be targeted for therapy.

## Discussion

In this study, we propose a computational method for conducting a differential flux analysis of metabolism at a genome-scale. This method allowed us to identify altered reactions in the metabolic network of NHBE cells infected with SARS-CoV-2 virus. This method comprises of a stringent statistical framework which enables us to filter out reactions which occur by chance due to the fundamental behaviour of linear optimization problems. This method is independent of objective function used and hence can be leveraged for understanding the metabolic flux alterations under given set of conditions in the case of mammalian cells.

Here, we leveraged flux sampling method ACHR (Artificial Coordinate Hit and Run)[31] to sample the flux solution space constrained by thermodynamic bounds and structure of the metabolic network. It is important to understand alternative methods like Flux Variability Analysis[30], which also helps us to probe into the differences in flux distribution in two conditions, assume a uniform distribution of fluxes across the solution space. This assumption makes it useful in allowing us to infer the maximum and minimum flux that a given reaction network can undertake in a given condition. Nevertheless, it fails to consider the likelihood of the actual flux distribution. This adds up to the limitations in inferring precise flux values from genome-scale metabolic reconstructions. Flux sampling analysis by the virtue of random sampling under metabolic and thermodynamic constraints[31, 48], provides a probability distribution function for the fluxes in their respective solution space. Thus, we can use statistical tests to obtain confidence intervals of the fluxes and compare them across multiple conditions. This would not have been otherwise possible if we assumed a uniform distribution of flux values in the solution space.

Kolmogorov-Smirnov test compares between the probability distribution of fluxes across several conditions and highlight metabolic modules that are altered. Furthermore, we use multiple-hypothesis testing to ensure the reported significances don’t contain false positives. The enriched set of reactions are further subjected to hypergeometric enrichment test for metabolic subsystems in the model. This is to make sure the reported pathways that are affected are not due to chance. Using this method, we find that several metabolic subsystems affected between diseased and normal state belong to fatty acid pathway in the case of SARS-CoV-2. Interestingly, there are already several reports which point at the severe deregulation in fatty acid metabolism confirmed at the level of clinical surveys under controlled trails and metabolomics studies[15, 40, 49]. In our analysis, we inform pathways such a fatty acid oxidation, arachidonic acid metabolism and beta-oxidation cycles to be severely affected apart from deregulations in amino acid and nucleotide metabolism pathways. The role of beta-oxidation in promoting viral pathogenesis has already been reported in several studies and we believe this could be important for therapeutic design[50]. Similarly, disturbances in arachidonic acid metabolism through the linoleic acid-arachidonic acid metabolism axis has been reported to be a hallmark of coronavirus infection[40].

Fatty acid are not only important energy precursors which might be expended by the virus to grow rapidly but also the biochemical precursor for several important signalling molecules. Leukotriene biosynthesis which begins from the oxidation of arachidonic acid has also been inferred to be one of the affected pathways between diseased and normal state. Leukotriene is an important autocrine and paracrine signalling molecule which allows cells to attract immune cells when abnormalities arise is homeostatic state of the cells[32]. It is possible that SARS-CoV-2 strategically interferes with such a signalling pathway by deregulating the biosynthesis pathway for the constituent signalling molecules. We were further interested in understanding how these metabolic changes are imparted on the normal cell by the virus. Metabolic fluxes are always under stringent regulation by the post-translational modifications or allosteric changes in the driver enzyme in shorter time-scales and by transcriptional regulation in longer time-scales. When SARS-CoV-2 infects the host cell, it is possible that viral proteins interfere with such regulatory controls and this allows the virus to reprogram the metabolism of the cells. A protein-protein interaction network between SARS-CoV-2 virus and the host cells has been published recently. We leveraged the same to check if the interactions of viral proteins with host proteins are enriched in the set of reactions we inferred from differential flux analysis. Surprisingly, we found several protein interactions and also phosphorylation changes in the enzymes subunits that are associated with altered flux. To infer the impact of protein interactions and phosphorylation on the enzyme functions, we used the Boolean relation between the enzyme subunits and the resulting functionality.

We would also like to mention some of the limitations of the current analysis and the corresponding pipeline. First, proteomics data would be more useful for constraining the metabolic models compared to transcriptomic data. But it wouldn’t make much difference to the current pipeline as tINIT has been extensively used with proteomics data[25]. It is imperative to choose a physiologically relevant objective function. In the case of simple unicellular microbes, a growth rate maximization objective can be chosen. The complex physiological objectives of mammalian cells or tissue prohibit the usage of such objective functions as in the case of microbes. It is also important to note that flux sampling is not deterministic and its predictive power will depend on how well the model is constrained. A poorly constrained model would show possible flux through multiple alternative pathways making it difficult to predict the one which is physiologically preferred. Therefore, sufficient data with respect to metabolite exchange rates must be considered while constraining the model. Given the power of multi-omics technologies, it will eventually becoming convenient to obtain metabolomics profiles and use them to constrain the model.

We consider this method to be a significant advancement in the field of metabolic flux analysis. This would allow us to probe into differences in flux apart from differences in network structure among several conditions. This would help us to design experiments to measure the effects of infections on specific metabolic modules in the cell. Such an analysis along with other multi-omics datasets like metabolomics, phospho-proteomics and interactomics will allow us to understand the molecular mechanistic principles of infections. Integration of multi-omics data is expected to reveal several mechanistic phenomena of diseases and will help us design better therapeutics leveraging the power of systems biology.

## Materials and methods

### Stoichiometric reconstruction of Virus Biomass Objective Function (VBOF) for SARS-CoV-2

All the calculations for nucleic acids, proteins, carbohydrates and lipids are conducted per gram dry weight (GDW) of the virus. The nucleotide content was calculated from the single stranded positive RNA sequence which is present as a single copy. The contribution of transcriptome towards nucleotide was also considered. Briefly, the number of transcripts per gram dry weight of the virus was calculated from the proteomics and translational efficiency data[34]. The translational efficiency is defined as the number of mRNA transcripts which are converted to polypeptides. The translational efficiency of various proteins were in the range ~ 0.1 to 2[34]. The highest efficiency was for the N protein while the lowest was for ORF1A and ORF3A. Nucleotides were the principal components of the biomass equation due to the high demands for mRNA synthesis apart from viral genome synthesis.

The contribution of amino acids to viral biomass was calculated from a proteomics dataset[41] published earlier. The copy number of spike (S) protein was considered as ~300 subunits per virion and the absolute stoichiometry of other proteins were calculated using it as a basis. It was important to validate the copy number of S protein on SARS-CoV-2. While it has been reported to approximately be the same as that for SARS Cov[33], it was imperative for us to validate it as VBOF calculation is directly dependent on that. We therefore leveraged electron micrographs of SARS-CoV-2 and used image analysis to estimate the copy number of spike protein on each virion. (S1 Fig and S1 Text).

N-acetyl Glucosamine (NAG) content of the biomass was calculated from the PDB file (PDB ID: 5SZS) of S protein. Briefly, the number of NAG molecules present in the crystal structure of a single S protein subunit was calculated. We obtained 63 NAG molecules per subunit of the virus in this way. The total number of NAG molecules studded on the S protein was calculated using the copy number information obtained above. The lipid composition was derived from previous studies[51]. Briefly, the total molecules of lipids present on the viral membrane was calculated from the surface area assuming 2 molecules per nm^2^ as discussed in earlier studies[52].

The total virus molecular weight was calculated from the sum of mass of all macromolecules present in the virus (S1 Dataset). The units of all the biomass precursors were converted to millimoles per GDW of SARS-CoV-2 using this information. The reconstructed biomass equation i.e. Cov2VBOF (SARS-CoV-2 Virus Biomass Objective Function) was added to HumanGEM model. Appropriate exchange reactions were added to enable flux through the VBOF. HAM media was used to constrain the uptake rates of substrates and oxygen in all the downstream analysis. The composition and substrate uptake rates are provided in S2 Dataset.

### Integration of gene expression data using tINIT

#### Gene expression data normalization

The gene expression data was derived from GSE147507. The triplicate data for mock infected NHBE cells and SARS-CoV-2 infected NHBE cells was considered. The information regarding the transcript isoform was obtained from Mammalian Transcriptomic Database (For Bronchial Epithelial Cells). The dataset was normalized to TPM (Transcripts per Million reads) using the transcript lengths specific for NHBE cells (S2 Dataset). Custom scripts were written in R for the same.

#### tINIT for integration of gene expression

The latest version of tINIT (version 2.0) was used to integrate the gene expression data into HumanGEM model[25]. The steps for reconstruction has been described in S2 Fig. COBRA Toolbox v3.0 was used for all metabolic flux analysis in MATLAB 2017b. We used Gurobi optimizer with the tINIT algorithm. Briefly, the highly curated HumanGEM model in a closed form i.e. all exchange reactions closed was fed to the program “getINITModel2” of RAVEN toolbox in MATLAB 2017b [53] along with the TPM normalized gene expression data. A gene expression cutoff of 1 TPM was used for the algorithm as reported earlier[25]. The integer optimization algorithm underlying tINIT tries to minimize the incorporation of transcripts below this level. The task list for tINIT was prepared as reported earlier[25]. The task list for the creation of context specific model of NHBE cells infected with SARS-CoV-2 has additional reactions (S2 Dataset). Apart from synthesizing non-essential amino acids, conduct oxidative phosphorylation and other basic metabolic tasks, the model was expected to conduct specialized tasks necessary for the growth of the virus. These additional reactions were the tasks to de-novo synthesize NAG needed for the synthesis of viral biomass and produce viral biomass i.e. Cov2VBOF from the components in the media (S2 Dataset). IBM CPLEX 12.1 (IBM Academic License) was used for running the tINIT program.

The resulting context specific models was constrained using the uptake rates from media components as reported in several literature (S2 Dataset). Flux Balance Analysis using either the HumanGEM biomass reaction or Cov2VBOF was used to validate the doubling time of the NHBE cells and SARS-CoV-2 respectively. This was compared to the experimentally measured data[41] and a t-test was conducted to show no significant differences between the experimental and theoretical growth rate for SARS-CoV-2.

Henceforth, we will call the context specific model for normal NHBE cells and SARS-CoV-2 infected NHBE cells as iNHBE and iNHBECov2. For calculation of Hamming distance, we constructed a reaction Boolean vector for each model. Briefly, we constructed a Boolean vector of length 13417 (corresponding to all the reactions in the base HumanGEM model) for each model with 1s and 0s i.e. whether a reaction from the base model was incorporated into the context-specific model or not.

#### VBOF sensitivity analysis

It was important check for the sensitivity of the specific growth rate prediction with respect to variations in coefficient (mmoles/gdw) or fractional composition of biomass precursors. We varied the coefficients of biomass precursors one at a time by ±10% and calculated the growth rate by flux balance analysis. The FBA was set-up on the modified SARS-CoV-2 biomass equations with coefficients increased by ±10% one at a time as follows:

$$maximize\;{c*}^{T}v$$

subjecttoi)S.v=0

ii)lowerbound≤v≤upperbound

Here, c* is the modified biomass coefficient vector such that c* = (c_**1**_**, c**_**2,**_ **c**_**3**_ **…. c**_**n**_**±0.1c**_**n**_**).** Here, the coefficient for c_n_ biomass precursor has been manipulated by ±10% whereas the coefficient of other biomass precursors remain the same.

IBM CPLEX solver was used to conduct the flux balance analysis. The context-specific model of NHBE infected with SARS-CoV-2 i.e. iNHBECov2 was considered for the analysis constrained with HAM media.

### Differential flux analysis

#### Flux sampling

ACHR (Artificial Co-ordinate Hit and Run) was used to sample fluxes from iNHBE and iNHBECov2. A thinning factor of 100 was used to obtain sparse and un-correlated fluxes which helps to span the whole flux space. IBM CPLEX was used for the optimization underlying ACHR flux sampling. The pipeline was executed in an i5 8th Generation Intel Core processor with 8 GB RAM. 10,000 sample points were drawn for each model from the sampling object. Custom scripts were written in Python 3.7 for the flux sampling estimation.

#### Determining differentially altered flux

Two-sample Kolmogorov Smirnov Test (KS Test) was used to differentiate between the flux distribution between iNHBE and iNHBECov2 models. A significance level of 0.05 was used for filtering the altered reactions. Moreover, we calculated the change in flux between two models from the sample means of the distributions. The flux mean in the case of infected and uninfected cells is the statistical mean of the flux distribution of each. S_infected_ and S_uninfected_ are the flux distributions for a given reactions in infected and uninfected conditions. The bar indicates the arithmetic mean of the distribution which is the most represented flux from the distributions. The flux change was calculated for each reaction as follows:

$$Flux\;Change\;(FC)=\frac{{\bar{S}}_{infected}-{\bar{S}}_{uninfected}}{\left|{\bar{S}}_{infected}+{\bar{S}}_{infected}\right|}$$

The denominator is used to normalize the magnitude of flux change. This is avoid biased filtering out of reactions with low flux but of important biological consequence. A Flux Change (FC) of 0.82 (corresponding to 10 fold change in flux) was used to filter reactions with insignificant flux change. For reactions exclusively present in one model, the flux through the same in the model where it is absent is assumed to be zero. To compare the flux distribution, we used bootstrapping to estimate the 95% confidence interval and then checked if zero is contained within the same. All reactions, where zero i.e. flux through the absent reaction, is outside the 95% confidence interval were considered to have a differential flux and taken for further analysis. A Benjamini-Hochberg FDR Multiple hypothesis correction with α = 0.05 was used to correct the p-values for all the resulting differentially altered reactions. The adjusted p-value was used along with the FC cutoff of 0.82 for filtering out the reactions which would also occur by chance.

#### Reaction enrichment analysis

Hypergeometric enrichment was used to obtain HumanGEM subsystems[25] (S2 Dataset) which are overrepresented in the altered set of reactions. Briefly, the enriched reaction list obtained from above was used to conduct a two-tailed hypergeometric test. Here, adjusted p-value was used to obtain the pathways showing significant representation based on the constituent altered reactions.

P(X=k)=(Kk)(N−Kn−k)(Nn)

Here, P(X = k) is the probability that we find k reactions just by chance in a given subsystem. K is the total number of reactions belonging to a given subsystem, N is the total number of reactions in the model and n is the total number of reactions that show altered flux as calculated by differential flux analysis. For each subsystem in the model, we conducted a hypergeometric enrichment test for overrepresentation. If P(X = k)<0.05, then it is likely that the over-representation of the subsystem is due to the high number of altered reactions in the pathway rather than by chance. The resulting p-values for pathways were again subjected to multiple-hypothesis correction using Benjamini-Hochberg method using False Discovery Rate (FDR) with α = 0.05. From this, we were able to obtain the pathways in the HumanGEM model which are most affected by the infection.

### Integrated Analysis of Protein-Protein Interaction Network (PPINs) and Differential Phosphorylation (DPs) with altered reactions

#### Reactions affected by PPINs and DPs

The PPIN dataset was obtained from recently published literature on host-virus interactome[46]. It is to be noted that the PPIN screen was conducted in a different cell line i.e. HEK-293T/17 cells. The differential phosphorylation data was obtained from another recently published report on phosphoproteomics[47]. Here, the datasets were either the interaction of viral proteins or phosphoproteome with the human gene products not with the protein complex. The association of the gene product with the activity of the protein complex can be presented by Boolean relation[23]. Similarly, in the case of metabolic model, the relationships between the gene product and the activity of enzyme is represented by a Boolean logic i.e. GPR (Gene-Protein-Reaction) association. Briefly, an OR relationship between two gene products represents isozymes i.e. Two alternative gene product that can provide the same activity. An AND relationship between two gene products implies that both the gene products are required to form an active enzyme complex. In order to map the effect of PPINs and DPs on the reactions, it was necessary to consider the Boolean relation. We used mapExpressionToReaction () within COBRA Toolbox v3.0 in MATLAB 2017b to map the effect on the gene product to the effect on the reactions. The gene products which were either associated with PPINs or DPs, we assign a value of 0 and the other non-interacting gene products were assigned a value of 1. If an enzyme is formed by complementation of two gene products i.e. AND relation, the reaction is affected even when just one of the gene products is associated with PPINs or DPs. But if the enzymatic activity is alternatively fulfilled by two separate gene products, the reaction is affected only when both of the gene products are associated with PPINs or DPs. Here, we consider PPINs and DPs are processes that influence activity of the gene products either through allosteric modifications or post-translational modifications. It is a characteristic nature of RNA virus to manipulate metabolism and regulation in the host mainly through short-term PPINs or other modifications rather than heavy transcriptional alteration. mapExpressionToReaction () yield a score for each reaction based on whether the reaction is affected (0) or unaffected(1) by the PPINs and DPs.

This set of reactions was compared to the set of reactions which were found to be differential altered in term of flux. A hypergeometric test was conducted to check if the enrichment of PPINs and DPs affected reactions in the set of reactions having differential flux were due to chance or not. The hypergeometric test was conducted as follows:

P(X=k)=(Kk)(N−Kn−k)(Nn)

Here, P(X = k) is the probability that we find k reactions affected by PPINs or DPs just by chance in a given subsystem. K is the total number of reactions affected by PPINs or DPs, N is the total number of reactions in the model and n is the total number of reactions that show altered flux as calculated by differential flux analysis. For each subsystem in the model, we conducted a hypergeometric enrichment test for overrepresentation. If P(X = k) <0.05, then it is likely that the over-representation of PPINs or DPs affected reactions are due to real effects of PPIN and DP on metabolism rather than by chance.

## Supporting information

S1 Fig The electron micrograph images of SARS Cov2 and calculation of spike protein count per virion from it. A) The electron micrographs of SARS-CoV-2 (Credit: NIAID-RML) B) Number of spike proteins calculated from intensity measurements of the micrograph along the circumference of the virus.
(TIF)

S2 Fig Pipeline for processing of RNA Seq data and tINIT for integration of gene expression data into the model. The processed final model is used for differential flux analysis.
(TIF)

S3 Fig Effect of change in uptake rates of nutrient on the fitness of the virus and NHBE cells. The uptake rates were varied and a point optimization program was used to calculate the specific growth rate. The fitness change is reported as the ratio of specific growth rate under perturbation to the specific growth rate under normal uptake rate. All the uptake rates were negative.
(TIF)

S4 Fig Sensitivity analysis of biomass objective function (VBOF) with respect to variations in biomass composition. The coefficients of biomass precursors were varied by ±10% taken one at a time and the specific growth rate was calculated by FBA.
(TIF)

S1 Dataset The reconstruction of SARS-CoV-2 biomass equation from the stoichiometric composition of the virus. The sheets in the dataset comprises of stepwise calculation of SARS-CoV-2 amino acid composition, nucleotide composition, carbohydrate and lipid composition. The last sheet comprises of the final viral biomass equation.
(XLSX)

S2 Dataset The datasets in S2 relate to various input files required for model reconstruction, the raw results from the analysis and other details. The description of each dataset within S2 Dataset are given in the first sheet.
(XLSX)

S1 Text Extended methods for derivation of spike protein copy number on SARS-CoV-2 using EM images and supplementary S1–S4 Figs. (PDF)
