# Supplementary figures and images for: Genome Scale-Differential Flux Analysis reveals deregulation of lung cell metabolism on SARS-CoV-2 infection
Source: PLoS Comput Biol. 2021 Apr 9;17(4):e1008860. doi: 10.1371/journal.pcbi.1008860 (PMC8034727; doi:10.1371/journal.pcbi.1008860)

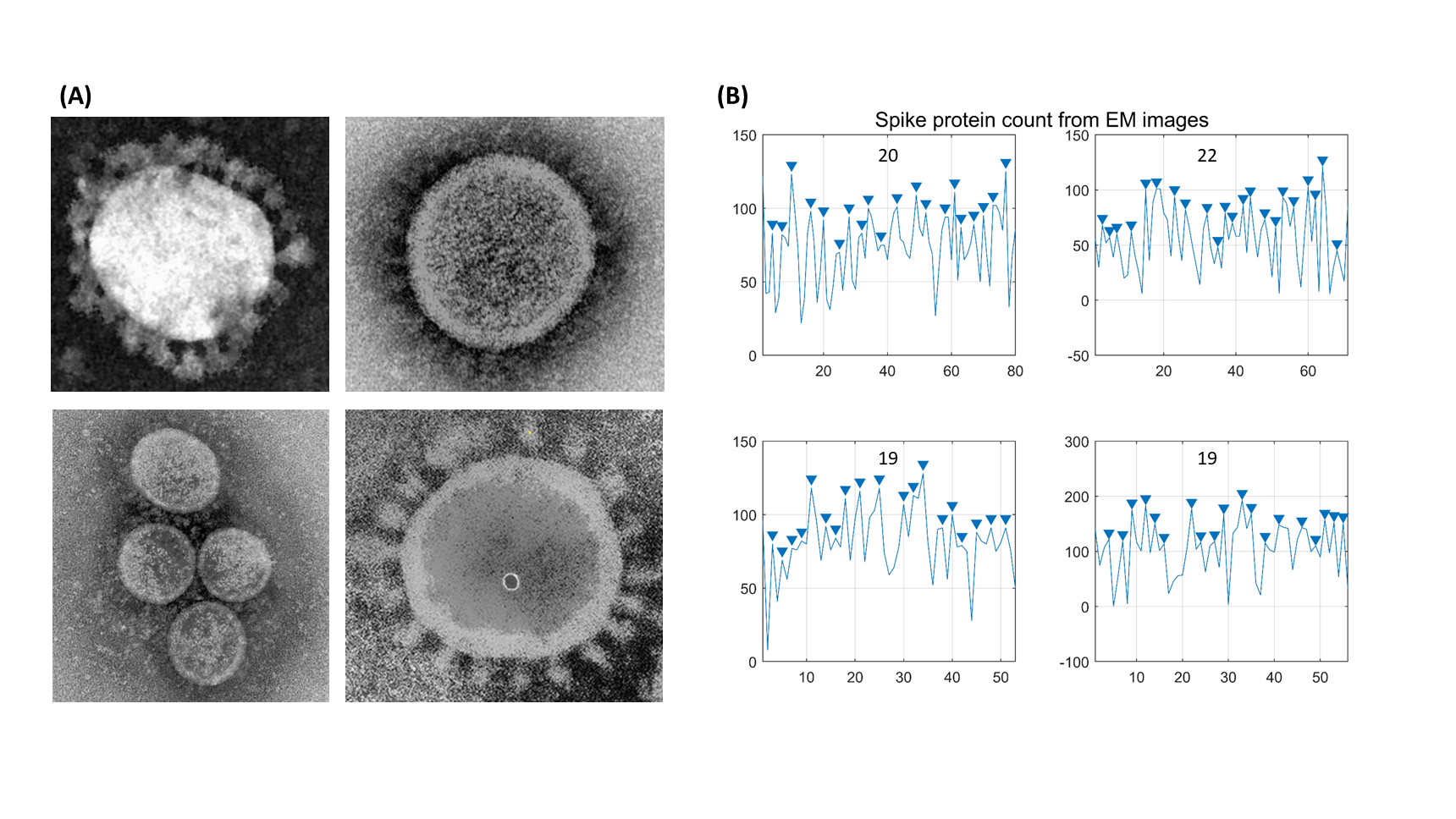

Supplement: S1 Fig — A) The electron micrographs of SARS-CoV-2 (Credit: NIAID-RML) B) Number of spike proteins calculated from intensity measurements of the micrograph along the circumference of the virus. (TIF) [file pcbi.1008860.s001.tif]

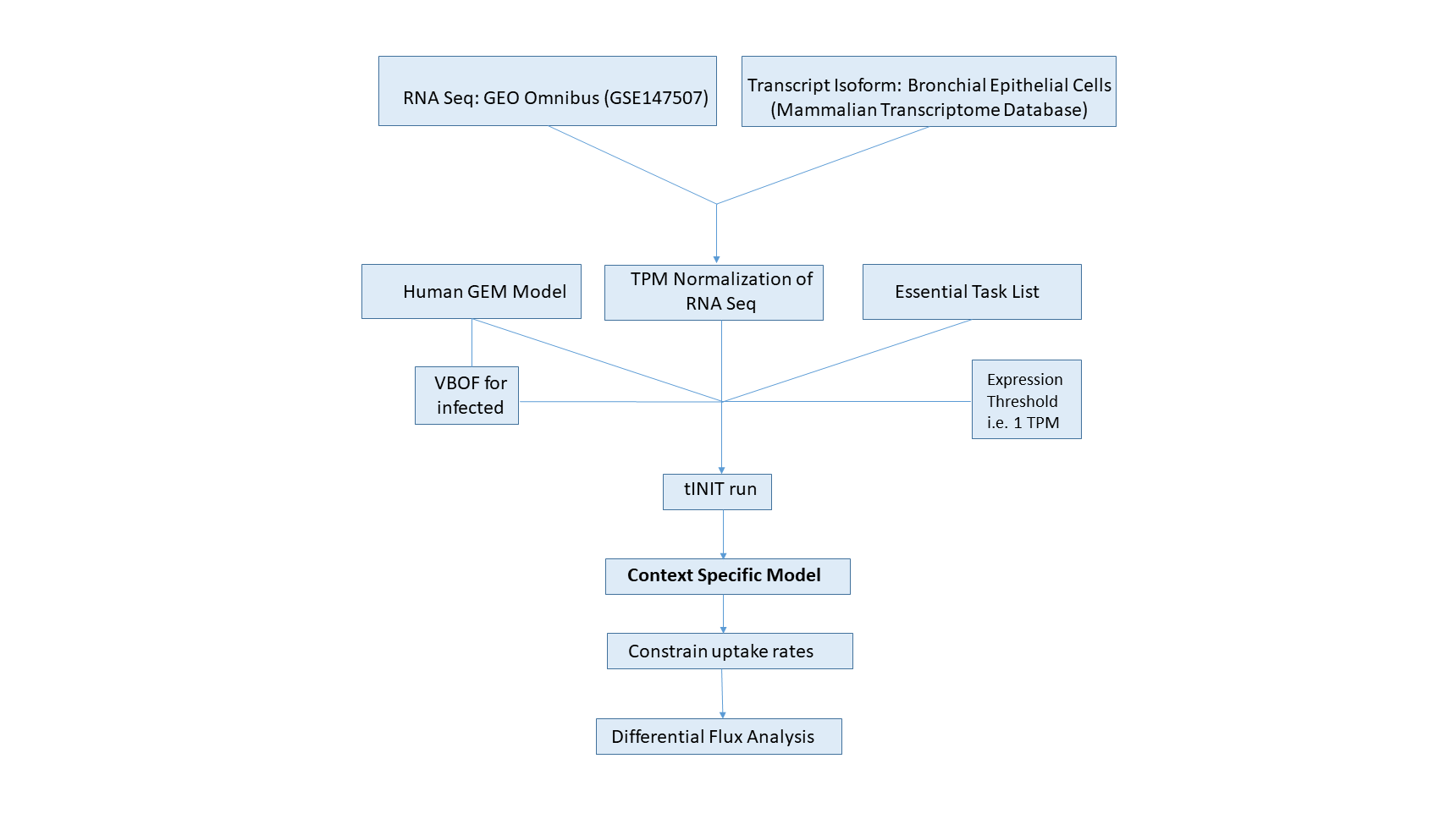

Supplement: S2 Fig — The processed final model is used for differential flux analysis. (TIF) [file pcbi.1008860.s002.tif]

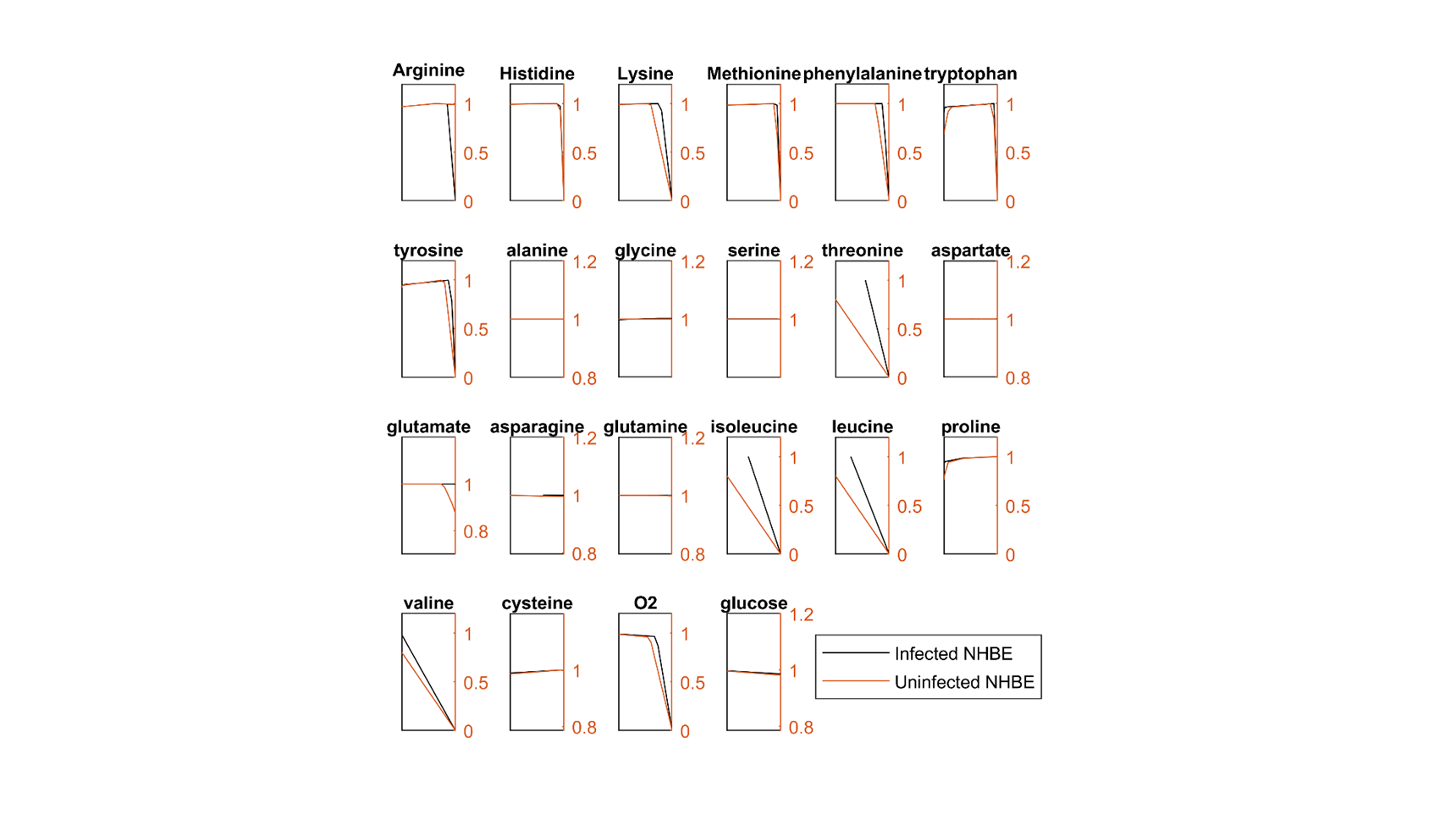

Supplement: S3 Fig — The uptake rates were varied and a point optimization program was used to calculate the specific growth rate. The fitness change is reported as the ratio of specific growth rate under perturbation to the specific growth rate under normal uptake rate. All the uptake rates were negative. (TIF) [file pcbi.1008860.s003.tif]

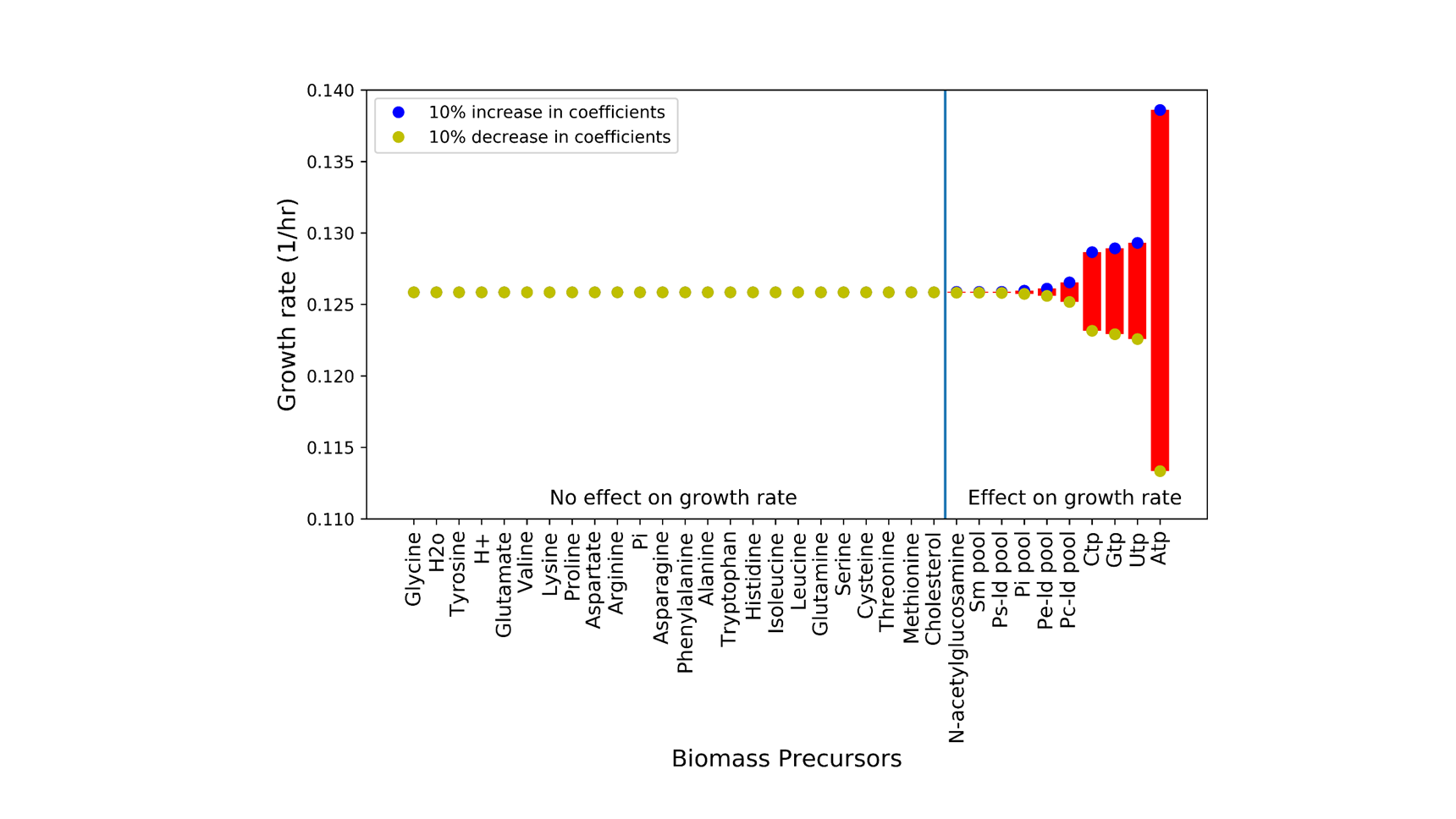

Supplement: S4 Fig — The coefficients of biomass precursors were varied by ±10% taken one at a time and the specific growth rate was calculated by FBA. (TIF) [file pcbi.1008860.s004.tif]
