# Supplementary material for: Genome Scale-Differential Flux Analysis reveals deregulation of lung cell metabolism on SARS-CoV-2 infection
Source: PLoS Comput Biol. 2021 Apr 9;17(4):e1008860. doi: 10.1371/journal.pcbi.1008860 (PMC8034727; doi:10.1371/journal.pcbi.1008860)
Supplement: S1 Text — (PDF) [file pcbi.1008860.s007.pdf]

# Supplementary Information for

## Genome Scale-Differential Flux Analysis (GS-DFA) reveals deregulation of lung cell metabolism on SARS Cov2 infection

Piyush Nanda[1], Amit Ghosh[2,3*]

*[1]Department of Biotechnology, Indian Institute of Technology Kharagpur, West Bengal, India-721302*
*[2]School of Energy Science and Engineering, Indian Institute of Technology Kharagpur, West Bengal, India-721302*
*[3]P.K. Sinha Centre for Bioenergy and Renewables, Indian Institute of Technology Kharagpur, West Bengal, India-721302*

*Corresponding author:
Dr. Amit Ghosh
Assistant Professor
School of Energy Science & Engineering
Indian Institute of Technology Kharagpur
Kharagpur 721302, West Bengal, India
Email: amitghosh@iitkgp.ac.in
Ph: +91-3222-260804

**Supplementary Information Text**

**Extended Methods:**

Estimating spike protein stoichiometry from electron microscopy images

The calculation of absolute protein numbers for SARS Cov2 is entirely based on the number of spike protein subunits on the virus. In recent discussions on the chemical composition of the virus(1), the spike protein subunit counts on SARS Cov2 has been assumed to be the same as that of SARS Cov. While this doesn't entirely defy logic, it would be imperative to validate the protein counts per virus in the SARS Cov2. For this purpose, we leveraged the electron micrographs of the virus taken by various research organizations. We used a combination of image analysis and mathematical modeling to derive the number of spike protein per virus.

Briefly, the electron micrographs were converted to 16 bit images. It could be observed that in several electron micrographs, the spike protein appear as the protrusions around the virus. We measured the intensities of multiple-points along the circumference of the virus. The number of spike proteins will be roughly equal to the number of positions along the circumference where we see high intensity values. We used ImageJ (FIJI) to estimate the intensity along the circumference of the virus at roughly uniformly placed points in order. It is to be noted that each intact spike protein comprises of 3 subunits. The plot between the intensity of various points (y-axis) and the angular position (x-axis) would give us intensity distribution at various angular positions. The number of peaks (corresponding to high intensity spots) would be roughly equal to the number of spike proteins (trimers) on the surface. We used custom codes written in MATLAB and leveraged 'findpeaks' functions to get the number of such peaks. This gave us the distribution of spike proteins (spike protein counts per circumference) in 2 dimensional cross section of the virus.

In order to estimate the distribution of spike proteins in the 3D surface of the virus, we assumed a uniform distribution of the spike protein on the surface. The following derivation was used to calculate the spike protein distribution (spike protein count per virus) on the surface:

N= Number of spike proteins calculated from the electron micrograph i.e. spike proteins along the circumference

n= Spike proteins per unit length circumference = $\frac{N}{2 \times \pi \times R}$

R = Radius of the cross section of the virus

Consider an elemental disk of width dx at a distance x from the center of the sphere.

Total number of spike protein on the elemental disk = $dn$ = Spike proteins along the circumference of the disk x Spike protein along the width of the disk

$$dn = \left[\frac{N}{2\times\pi\times R}\times 2\times\pi\times\sqrt{R^2-x^2}\right]\times\left[\frac{N}{2\times\pi\times R}\times dx\right]$$

The integration of *dn* while x increases from 0 to R will give us the distribution of spike proteins in one hemisphere of the circle.

Therefore,

Let $N_{total}$ = Total number of spike protein on the surface

$$0.5\times N_{total} = \int_0^R dn$$

$$0.5\times N_{total} = \int_0^R \left[\frac{N}{2\times\pi\times R}\times 2\times\pi\times\sqrt{R^2-x^2}\right]\times\left[\frac{N}{2\times\pi\times R}\times dx\right]$$

$$0.5\times N_{total} = \frac{N^2}{8}$$

$$N_{total} = \frac{N^2}{4} \quad ..(i)$$

Figure S1 shows the number of spike proteins calculated from the electron micrographs (N). The average N i.e. <N> ~ 20. Substituting that in (i), we get $N_{total}$ ~ 100. Note that $N_{total}$ is the total number of spike trimers, so the total number of spike subunits on the surface is ~300 which is the same reported for SARS Cov in recent discussions.

Hence, this analysis provides direct evidence of spike protein counts on the surface of the virus and validates the assumption. We can therefore use this estimated protein count for generation of biomass objective function/biomass equation for the SARS Cov2 virus.

## References

1. Bar-on YM, Flamholz A, Phillips R, Milo R. SARS-CoV-2 ( COVID-19 ) by the numbers. Elife. 2020;
